# Supplementary material for: Targeted Integration of Inducible Caspase-9 in Human iPSCs Allows Efficient in vitro Clearance of iPSCs and iPSC-Macrophages
Source: Int J Mol Sci. 2020 Apr 3;21(7):2481. doi: 10.3390/ijms21072481 (PMC7177583; doi:10.3390/ijms21072481)
Supplement: Supplementary file 1 [file ijms-21-02481-s001.zip › Supplementary table 1.docx]

| \| **Supplementary table 1. Secondary culture of iPSCs after exposure to AP20187** \| \| --- \| | | | | | | | |
| --- | --- | --- | --- | --- | --- | --- | --- | --- |
| AP20187 [nM] | | CD34iPSC16exi | | iCasp9-mono | | iCasp9-bi | |
| 0 | Confl. | | Confl. | Confl. | Confl. | Confl. | Confl. |
| 1 | Confl. | | Confl. | 0 | 0 | 0 | 0 |

| Confl.- cells growing to confluency within 14 days |
| --- |
