# Supplementary figures and images for: Targeted Integration of Inducible Caspase-9 in Human iPSCs Allows Efficient in vitro Clearance of iPSCs and iPSC-Macrophages
Source: Int J Mol Sci. 2020 Apr 3;21(7):2481. doi: 10.3390/ijms21072481 (PMC7177583; doi:10.3390/ijms21072481)

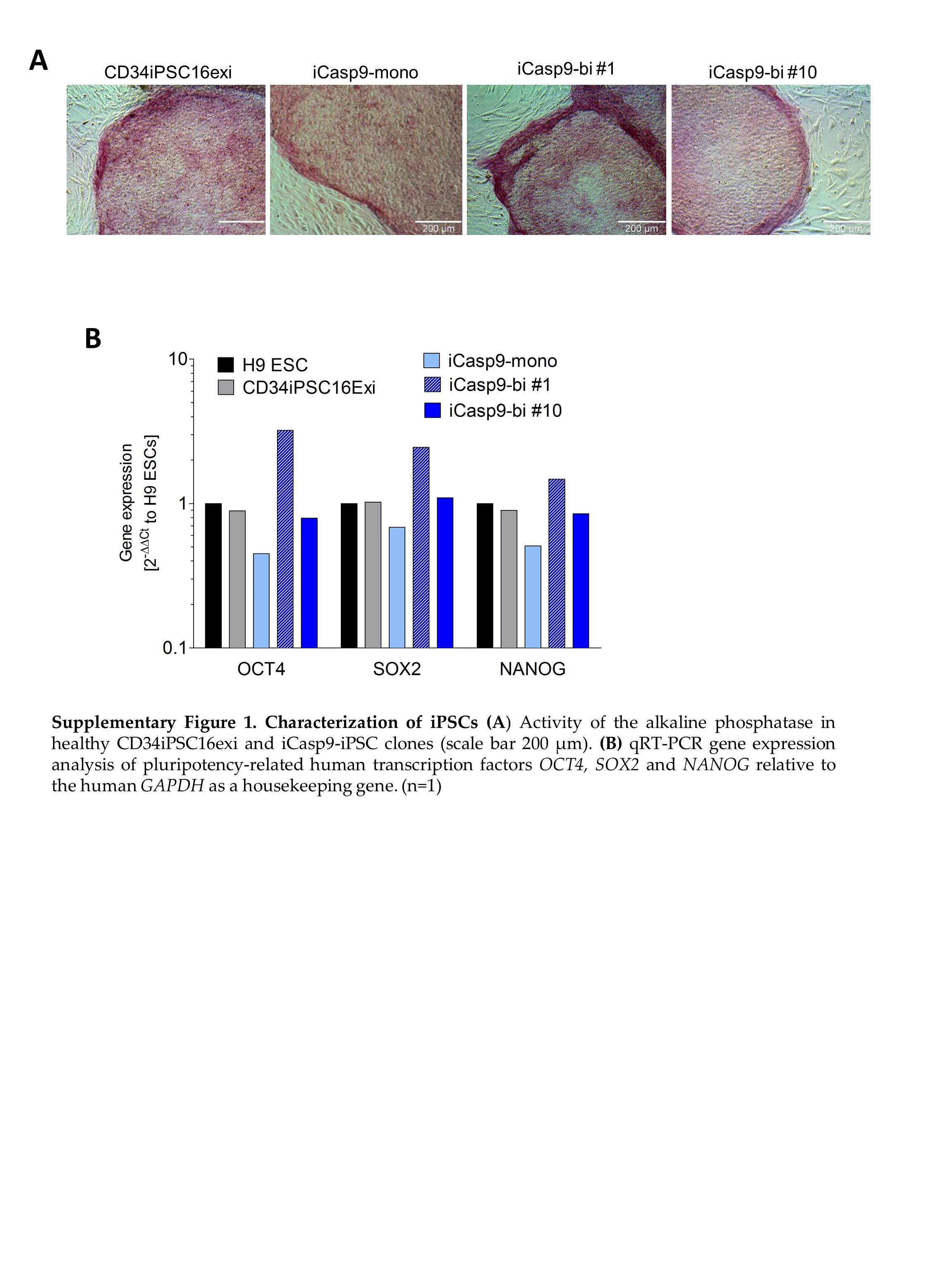

Supplement: Supplementary file 1 [file ijms-21-02481-s001.zip › S1.jpg]

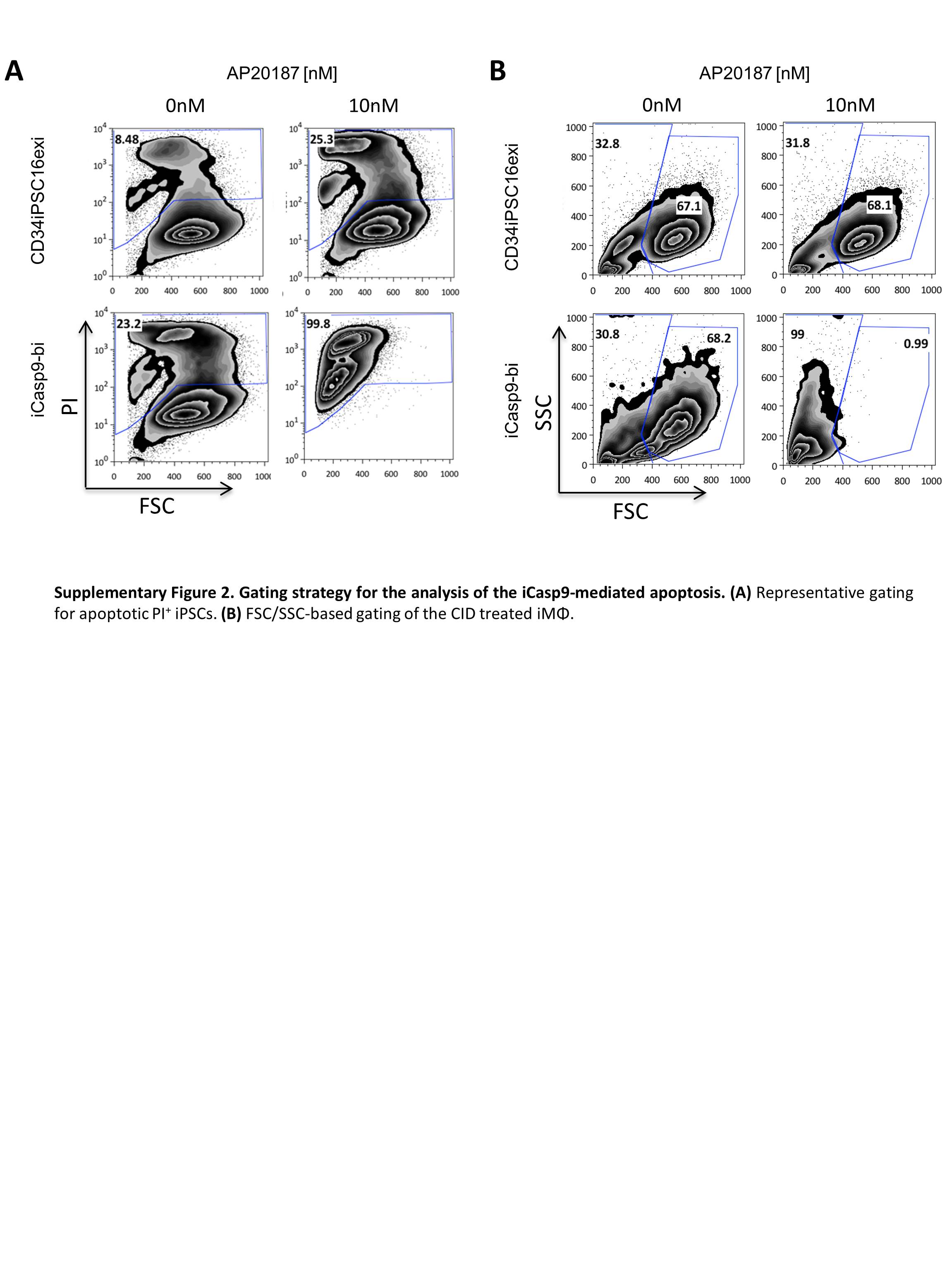

Supplement: Supplementary file 1 [file ijms-21-02481-s001.zip › S2.jpg]

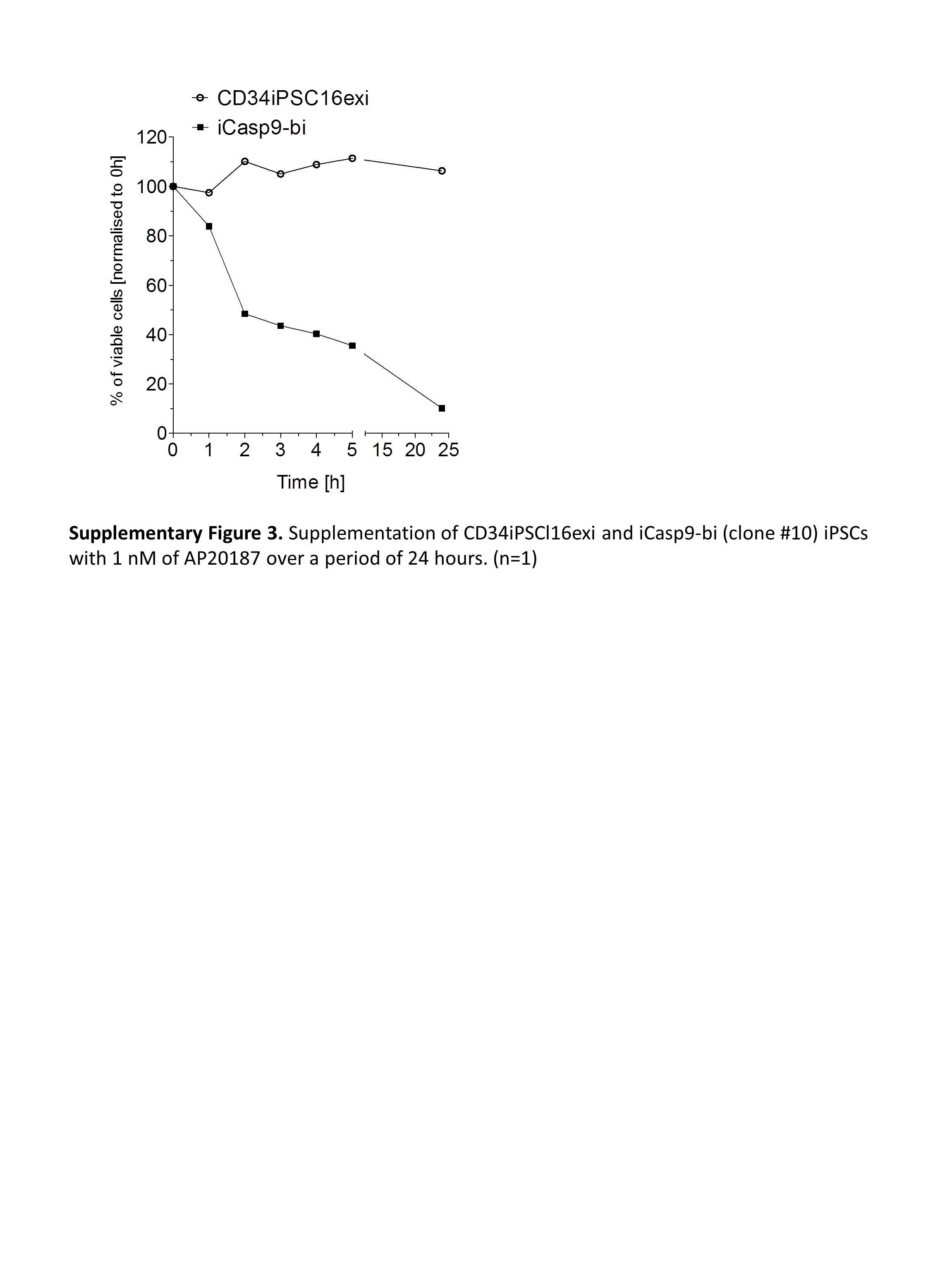

Supplement: Supplementary file 1 [file ijms-21-02481-s001.zip › S3.jpg]
